# Defective Expression of Scavenger Receptors in Celiac Disease Mucosa

**DOI:** 10.1371/journal.pone.0100980

**Published:** 2014-06-27

**Authors:** Maria Laura Cupi, Massimiliano Sarra, Daniela De Nitto, Eleonora Franzè, Irene Marafini, Ivan Monteleone, Giovanna Del Vecchio Blanco, Omero Alessandro Paoluzi, Davide Di Fusco, Paolo Gentileschi, Angela Ortenzi, Alfredo Colantoni, Francesco Pallone, Giovanni Monteleone

**Affiliations:** Dipartimento di Medicina dei Sistemi, Università Tor Vergata, Rome, Italy; Massachusetts General Hospital and Harvard Medical School, United States of America

## Abstract

Celiac disease (CD) is a gluten sensitive enteropathy characterized by a marked infiltration of the mucosa with immune cells, over-production of inflammatory cytokines and epithelial cell damage. The factors/mechanisms that sustain and amplify the ongoing mucosal inflammation in CD are not however fully understood. Here, we have examined whether in CD there is a defective clearance of apoptotic cells/bodies, a phenomenon that helps promote tolerogenic signals thus liming pathogenic responses. Accumulation of apoptotic cells and bodies was more pronounced in the epithelial and lamina propria compartments of active CD patients as compared to inactive CD patients and normal controls. Expression of scavenger receptors, which are involved in the clearance of apoptotic cells/bodies, namely thrombospondin (TSP)-1, CD36 and CD61, was significantly reduced in active CD as compared to inactive CD and normal mucosal samples. Consistently, lamina propria mononuclear cells (LPMC) of active CD patients had diminished ability to phagocyte apoptotic cells. Interleukin (IL)-15, IL-21 and interferon-γ, cytokines over-produced in active CD, inhibited the expression of TSP-1, CD36, and CD61 in normal intestinal LPMC. These results indicate that CD-related inflammation is marked by diminished clearance of apoptotic cells/bodies, thus suggesting a role for such a defect in the ongoing mucosal inflammation in this disorder.

## Introduction

Celiac disease (CD) is a chronic enteropathy that occurs in genetically-predisposed individuals following ingestion of gluten proteins of wheat, rye, and barley. Histologically, CD is characterized by various grades of epithelial damage and atrophy of the small intestinal villi, hyperplasia of the crypts and a marked infiltration of the lamina propria and intra-epithelial compartments with inflammatory cells [1], [2].

The only accepted treatment for CD is a lifelong gluten-free diet (GFD), which results in complete remission of symptoms and recovery of the normal mucosal histology [2]. Recent studies have delineated some of the mechanisms by which gluten triggers the CD-related pathogenic response. For example, it has been demonstrated that gluten peptides can activate both innate and adaptive immune cells with the downstream effect of producing a vast array of inflammatory cytokines [3], [4]. One such cytokine is interleukin (IL)-15, which activates antigen-non specific CD8+ T cells and NK cells and hence facilitates epithelial cell damage [5]. IL-15 can also target lamina propria CD4+ cells and increase synthesis of IL-21 [4], [6]. Gluten-specific CD4+ T cells produce elevated levels of interferon (IFN)-γ, that is supposed to play a decisive role in the pathological process leading to tissue damage [6], [7], [8]. Moreover, in CD, there is enhanced production of IL-17A by gluten non-reactive T cells, thus raising the possibility that additional factors other than gluten are involved in the amplification and perpetuation of the ongoing mucosal inflammation in this disorder [9], [10]. There is also evidence that CD-related inflammation is marked by increased apoptosis of immune cells and enterocytes, suggesting that mucosal flattening is a consequence of exaggerated epithelial cell death [11], [12], [13].

In physiologic conditions, elimination of apoptosing cells or apoptotic bodies by scavengers (e.g. macrophages/dendritic cells) prevents secondary necrosis and promotes tolerogenic responses [14], [15], [16]. The interaction between the phagocytes and apoptotic cells is mediated by a variety of macrophage membrane-associated proteins (e.g. CD36, CD61, CD91) [15], [17]. Interaction of CD36 with apoptotic cells is mediated specifically by thrombospondin-1 (TSP-1), an extracellular matrix glycoprotein that bridges apoptotic cells, CD36 and CD61, thus creating a phagocytically active ternary complex [18], [19]. When massive apoptosis overwhelms the available scavenging capacity or when the scavenger mechanism is impaired, the secondary necrosis ensues thereby leading to a deregulated inflammatory response and tissue injury [14].

The aim of this study is to examine whether CD-associated inflammation is marked by changes in the levels of scavenger receptors and dysfunctional apoptotic cell clearance.

## Materials and Methods

### Ethics Statement

Written informed consent was obtained from all patients, controls and healthy volunteer donors and the study protocol was approved by the Ethics Committee Policlinico Universitario Tor Vergata, Rome, Italy.

### Mucosal samples

Duodenal biopsies were taken from 15 patients with active CD (ACD) during upper gastrointestinal endoscopy. The histopathological diagnosis of CD was based on typical lesions characterized by villous atrophy and crypt cell hyperplasia. All ACD patients were positive for anti-endomysial (EMA) and anti-transglutaminase-2 (anti-TG2) antibodies at the time of diagnosis. Biopsies were also taken from 9 inactive CD (ICD) patients who were on a strict GFD for at least 1 year. The ICD patients were in clinical and histological remission and negative for EMA and anti-TG2. Duodenal biopsies were also available from 12 non-CD controls (NC), who were under investigation for gastrointestinal symptoms, but had normal histology and were EMA and anti-TG2 negative. Normal jejunal specimens, taken from individuals undergoing gastro-jejunal bypass for obesity, were used for isolating lamina propria mononuclear cells (LPMC).

### Tunel Assay

Apoptosis was evaluated on frozen sections of human duodenal biopsies using the TUNEL assay (ApopTag Plus Peroxidase In Situ Apoptosis Detection Kit, Millipore, Billerica, MA) according to the manufacturer’s instruction.

### RNA extraction, complementary DNA preparation, and real-time PCR

RNA was extracted by using TRIzol reagent (Life Technologies, Monza, Italy) according to the manufacturer’s instructions. A constant amount of RNA (0.5 µg per sample) was retro-transcribed into complementary DNA, and this was then amplified using a SYBR-green Master mix (Bio-Rad, Milan, Italy) with the following conditions: denaturation for 1 min at 95°C, annealing for 30 s at 60°C for β-actin and 61°C for CD61, followed by 30 s of extension at 72°C. Primer sequence was as follows: β-actin FWD: 5′-AAGATGACCCAGATCATGTTTGAGACC-3′ and REV: 5′-AGCCAGTCCAGACGCAGGAT-3′; CD61 FWD: 5′-CCTGTATGTGGTAGAAGAGCC-3′ and REV: 5′-TTTCGGTCGTGCATGGTGATG-3. Human TSP-1, CD36 and CD91 were evaluated using commercially available TaqMan probe (Life Technologies, Monza, Italy). β-actin was used as housekeeping gene and gene expression was calculated using the ΔΔCt algorithm.

### Cell isolation and culture and phagocytosis assay

All reagents were from Sigma-Aldrich (Milan, Italy) unless specified. LPMC were isolated from normal jejunal surgical specimens as previously described [9] and resuspended in RPMI-1640 supplemented with 10% fetal bovine serum, penicillin (100 U ml^−1^), streptomycin (100 µg ml^−1^), and gentamycin (50 µg ml^−1^) (complete medium; Lonza, Milan, Italy), and cultured with or without IL-15 (50 ng/ml, R&D systems), IL-21 (100 ng/ml; Life technologies, Milan, Italy) or IFN-γ (100 ng/ml; Peprotech, Hamburg, Germany). After 18 hours, cells were used to extract RNA.

Human peripheral blood mononuclear cells (PBMC) were isolated from enriched buffy coats of healthy volunteer donors by Ficoll gradients and used to purify CD14+ cells by magnetic beads according to the manufacturer’s instruction (MiltenyiBiotec; Bergish Gladbach, Germany). CD14+ cells were labeled with CM-Dil (Life Technologies, Monza, Italy) and cultured in RPMI 1640 without serum in the presence of staurosporine (50 ng/ml) for 30 minutes to induce apoptosis. Apoptosis was evaluated by flow-cytometry using a commercially available antibody for Annexin V (Immunotools GmbH, Friesoythe, Germany) and Propidium Iodide (Sigma-Aldrich).

To analyze the ability of LPMC to phagocytise apoptotic bodies, LPMC isolated from 4 ACD patients, 3 ICD patients and 4 NC patients, were labelled with carboxyfluorescein-diacetatesuccinimidyl ester (CFSE) and incubated with CM-Dil-positive apoptotic CD14+ monocytes (ratio 1∶4) for 5 hours. The percentage of CFSE/CM-Dil-double positive cells was assessed by flow cytometry (FACScalibur, BD Bioscience, San Jose, CA).

### Immunohistochemistry and immunofluorescence

Frozen sections of duodenal biopsy samples were stained with mouse anti-human CD36 (1∶200 final dilution, BD Bioscience, San Jose, CA) followed by incubation with alexafluor 488-conjugatedsecondary antibody (1∶400 final dilution, Life Technologies). Additional sections were fixed with paraformaldehyde and ethanol, and incubated with a mouse anti-human TSP-1 antibody (1∶100 final dilution, Novus Biologicals, Cambridge, UK) or mouse anti-human CD61 (1∶8000 final dilution, BD Bioscience, San Jose, CA) for 1 h at room temperature. Immunoreactive cells were visualized using Ultravision HRP-Polymer kit (Thermo Fisher Scientific, Waltham, MA) and DAB (Dako, Glostrup, Denmark), according to the manufacturer’s instructions, and lightly counterstained with hematoxylin. Control sections were prepared under identical immunohistochemical conditions replacing the primary antibody with isotype control antibodies (Dako).

### Statistical analysis

Differences between groups were compared using the Student t test or Mann-Withney U test depending of the data distribution.

## Results

### Celiac disease-related inflammation is marked by accumulation of apoptotic cells/bodies and defective expression of scavenger receptors

**Figure 1:**
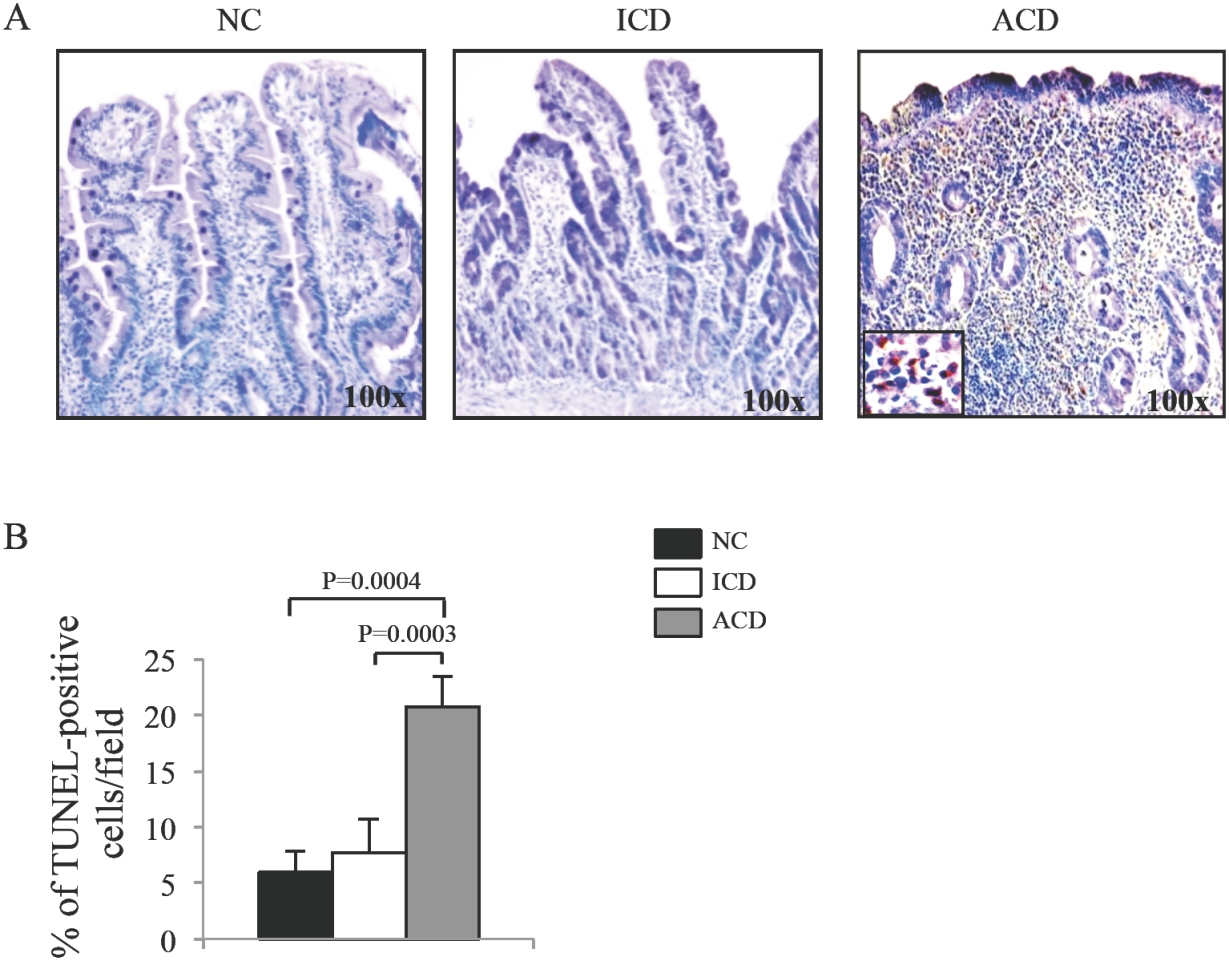
Celiac disease-related inflammation is marked by mucosal accumulation of apoptotic cells/bodies. A. Representative photomicrographs (original magnification 100x) of TUNEL-stained paraffin-embedded sections of duodenal samples taken from 1 normal control (NC), 1 inactive celiac disease (ICD) patient and 1 active celiac disease (ACD) patient. B. TUNEL-positive cells were counted in at least 5 fields/section of duodenal samples of 3 NC, 3 ICD and 3 ACD patients. Data indicate mean ± SD.

A small number of apoptotic cells/bodies was evident in NC and ICD samples, particularly in the epithelial compartment (Fig. 1A). In contrast, in ACD mucosa there was a significant increase in the number of apoptotic cells/bodies as compared to both NC and ICD and this was evident in both epithelial and lamina propria compartments (Fig. 1A–B).

**Figure 2:**
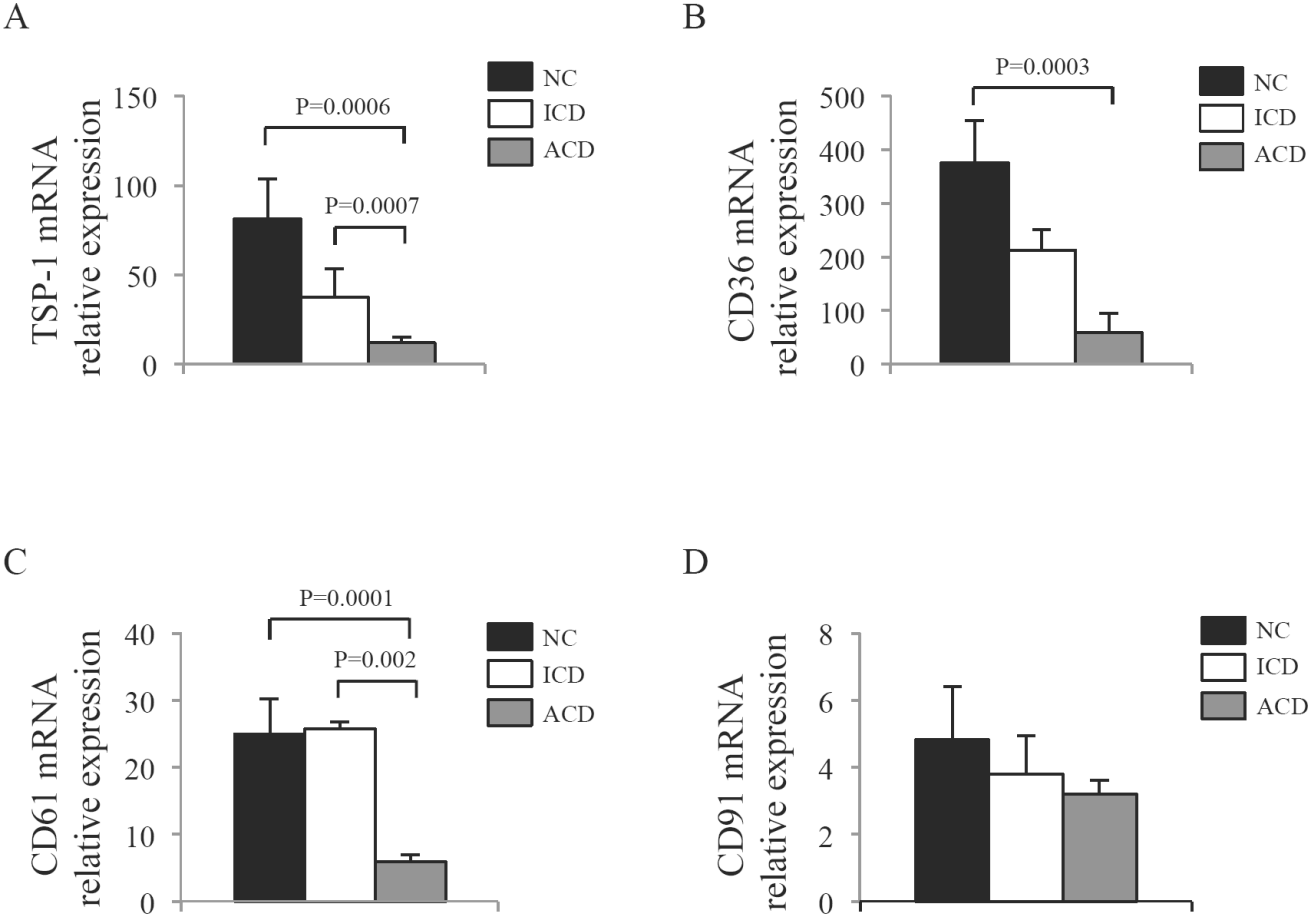
Reduced expression of scavenger-associated markers in celiac disease. Duodenal biopsy samples were taken from 12 normal controls (NC), 9 inactive celiac disease (ICD) patients and 15 active celiac disease (ACD) patients and analyzed for TSP-1 (A), CD36 (B), CD61 (C) and CD91 (D) RNA expression by Real-Time PCR, PCR and levels were normalized to β-actin. Data indicate mean ± SEM of all experiments.

**Figure 3:**
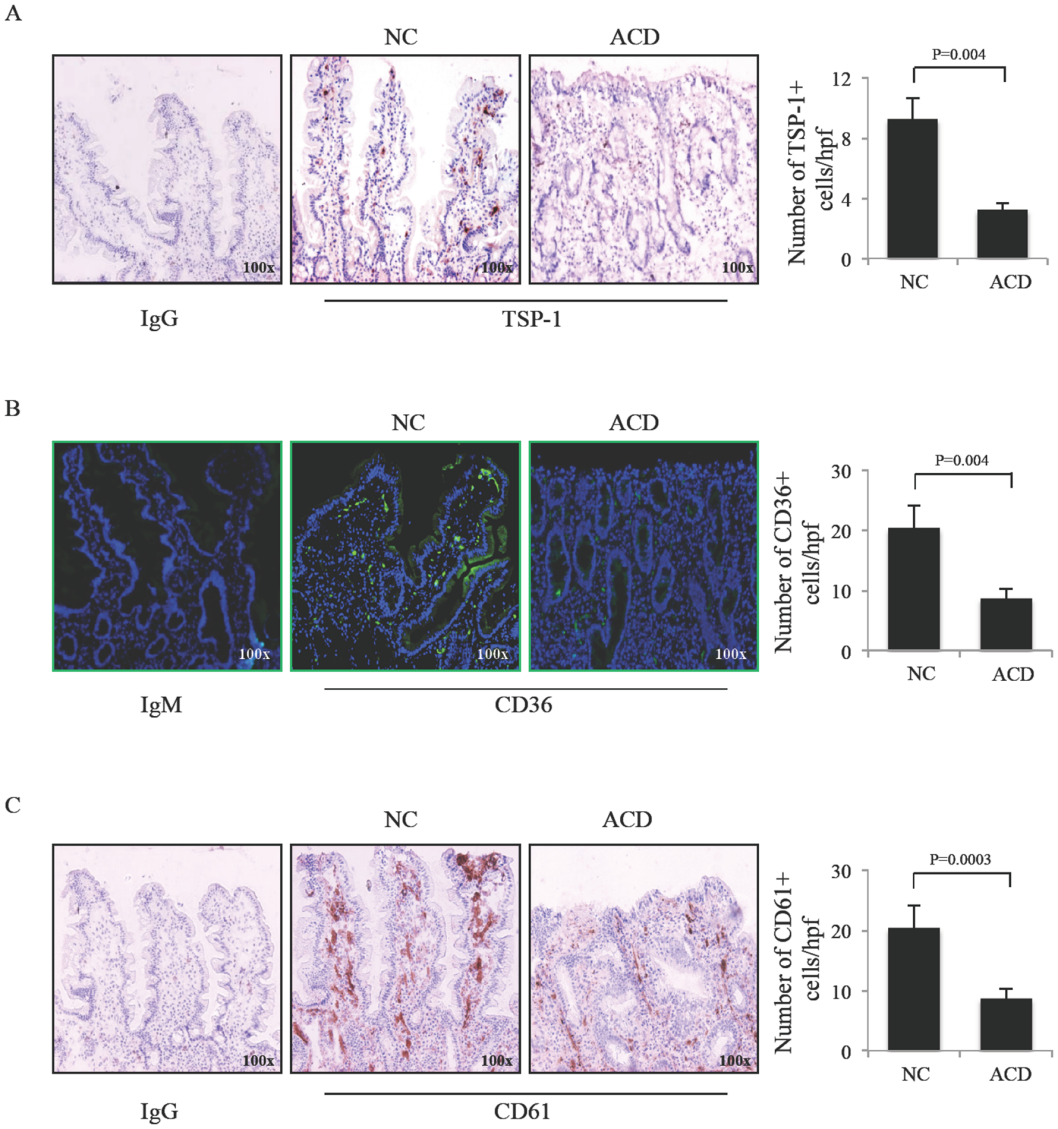
Thrombospondin-1, CD36, and CD61 protein expression is down-regulated in active celiac disease. Representative photomicrographs showing Thrombospondin-1 (A), CD36 (B) and CD61 (C) immunostaining in duodenal sections of 1 non-CD controls (NC) and 1 active celiac disease (ACD) patient. Isotype control staining is also shown. Right panels: positive cells were counted in at least 5 fields/section of duodenal samples of 5 NC and 5 ACD patients and expressed as mean ± SD of all experiments.

To determine whether the accumulation of apoptotic cells/bodies in ACD was associated with changes in the expression of scavenger receptors, we initially analyzed RNA transcripts of TSP-1, CD36, CD61 and CD91 by real-time PCR. RNA expression of TSP-1 and CD61 was significantly reduced in ACD samples as compared to NC and ICD, while there was no significant difference between ICD and NC (Fig. 2A–C). Moreover, a significant down-regulation of CD36 transcripts occurred in ACD as compared to NC, with no significant difference between ACD and ICD, and ICD and NC (Fig. 2B). In contrast, CD91 RNA transcripts were expressed at similar levels in the 3 groups (Fig. 2D). To confirm the reduced expression of scavenger markers in ACD, duodenal sections of NC and ACD patients were stained with anti-CD36, anti-TSP-1 and anti-CD61 antibody. The number of TSP-1- (figure 3A) CD36- (figure 3B), and CD61-positive (figure 3C) cells in the duodenal lamina propria of ACD was diminished as compared to NC.

### CD is associated with a defective ability of LPMC to phagocyte apoptotic bodies

**Figure 4:**
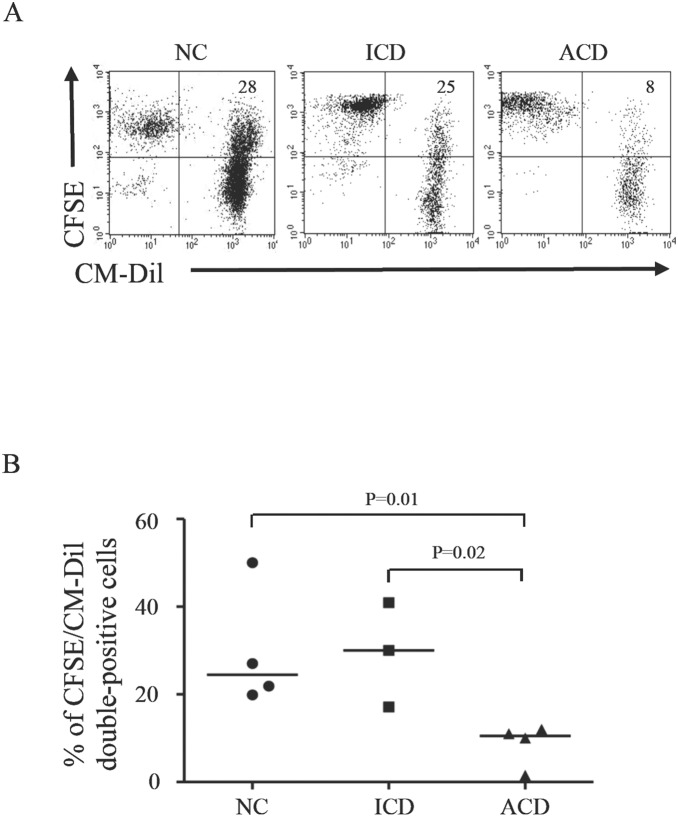
Intestinal lamina propria mononuclear cells (LPMC) isolated from celiac disease (CD) patients have diminished phagocytic activity. A. LPMC, isolated from biopsy samples of 4 active CD (ACD), 3 inactive CD (ICD) and 4 non-CD controls (NC), were labelled with CFSE and cultured with CM-Dil-positive apoptotic monocytes. After 5 hours, CFSE/CM-Dil double-positive cells were evaluated by flow-cytometry. Representative dot plots show CFSE-labelled LPMC taking-up CM-Dil apoptotic monocytes. Numbers in the quadrants indicate the percentage of CFSE/CM-Dil double-positive cells. B. Percentages of CFSE-labelled LPMC taking-up CM-Dil apoptotic monocytes. Each point in the graph indicates the percentage of double-positive cells in a single sample of a single patient. The horizontal bars represent the median values.

Next, we determined whether the reduced expression of scavenger receptors in ACD associates with defective ability of LPMC to take-up apoptotic bodies. As a standard model of apoptotic cells, we used circulating monocytes induced to become apoptotic by culture in serum-free medium and exposure to staurosporine. LPMC were isolated from duodenal biopsy sample of ACD patients, ICD patients and NC, labeled with CFSE and cultured with CM-Dil-positive monocyte-derived apoptotic cells. The representative dot-plots in figure 4A show that nearly one fourth of CFSE-labelled LPMC of NC and ICD and less than 10% of those in ACD co-expressed CM-Dil, thus indicating that such cells had phagocytic activity. The percentage of CFSE/CM-Dil double-positive LPMC in ACD was significantly reduced as compared to NC and ICD (Fig. 4B).

### IL-15, IL-21 and IFN-γ differently modulate expression of CD36, CD61 and TSP-1 in normal LPMC

**Figure 5:**
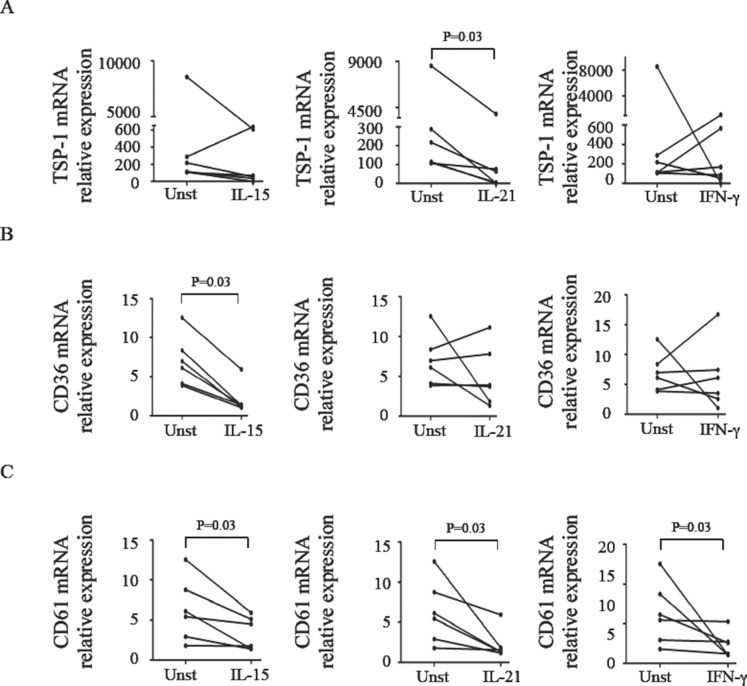
IL-15, IL-21 and IFN-γ modulate expression of TSP-1, CD36, and CD61 in normal LPMC. LPMC isolated from the jejunal mucosa of 6 normal controls were cultured with or without (Unst = unstimulated) IL-15 (50 ng/ml), IL-21 (50 ng/ml) and IFN-γ (100 ng/ml) and then analyzed for TSP-1 (A), CD36 (B), and CD61 (C) RNA expression by Real-Time PCR and levels were normalized to β-actin. Data indicate mean ± SD of all experiments.

CD-related inflammation is associated with enhanced production of IL-15, IL-21 and IFN-γ [7], [8], [10]. Therefore, we next determined whether such cytokines inhibit expression of CD36, CD61 and TSP-1 in normal LPMC. Since the number of cells isolated from pinch endoscopic biopsies is not always sufficient to carry out functional studies, we isolated normal LPMC from jejunal specimens of individuals undergoing intestinal by-pass for obesity. Treatment of LPMC with IL-15 significantly reduced CD36 and CD61, but not TSP1, RNA transcripts (Fig. 5). IL-21 significantly inhibited TSP-1 and CD61 RNA expression leaving unchanged CD36 transcripts, while IFN-γ reduced only CD61 transcripts (Fig. 5).

## Discussion

The findings of the present study demonstrate that the active phases of CD are characterized by accumulation of apoptotic cells/bodies in the duodenal mucosa, thus confirming data of previous studies [11], [12], [13], and diminished expression of macrophage-associated scavenger receptors, which are important in the recognition of exposed phosphatidylserine on the surface of apoptotic cells. In particular, we show that ACD patients have reduced mucosal levels of CD36, TSP-1 and CD61, also known as ανβ3 integrin or CD51 or vitronectin receptor, which altogether constitute an active complex mediating phagocytosis of apoptotic bodies/debris. In contrast, the levels of CD91 in ACD do not differ from those in ICD and NC. Our data are consistent with studies in chronic obstructive pulmonary disease showing that the defective ability of alveolar macrophages to phagocyte apoptotic cells is associated with a smoking-related reduction of scavenger receptors [20], [21].

Multiple receptors can be used by macrophages to ingest apoptotic cells, and the type of receptor involved in such a process depends primarily on the activation state of the macrophage rather than on its species or site of origin or the specific apoptotic target cell [22]. Therefore, we next determined whether, in ACD, the diminished expression of scavenger receptors associated with diminished phagocytic activity of LPMC. Our data indicate that LPMC isolated from the inflamed gut of ACD patients are markedly deficient in ability to ingest apoptotic cells in vitro. In theory, the diminished phagocytic activity of ACD LPMC could rely on other mechanisms rather than the relative deficiency of scavenger receptor. One possibility is that ACD LPMC could actually ingest and digest apoptotic bodies so rapidly that they could not be detected in vitro. This hypothesis appears however unlikely as analysis of the phagocytic activity of LPMC was performed after 5 hours of culture, a time point which is too short to allow cells to take-up and digest apoptotic cells. Another possibility is that the in vitro ingestion of apoptotic cells by ACD LPMC is down-regulated due to exaggerated ingestion of digestible particles or apoptotic cells occurring in the gut of ACD patients. This possibility is suggested by studies in other systems showing that rat bone-marrow derived macrophages exhibit a reduced ability to phagocyte apoptotic neutrophils after an initial round of phagocytosis [23]. However, we reject this hypothesis as the marked accumulation of apoptotic cells/bodies in the inflamed gut of ACD patients suggests the defective rather than excessive clearing of apoptotic cells by mucosal macrophages occurs in this disease.

The factors/mechanisms underlying the reduced expression of CD36, TSP-1 and CD61 in ACD remain to be clarified. Functional studies with normal LPMC indicated however that cytokines over-produced in ACD may be involved as stimulation of such cells with IL-15, IL-21 and IFN-γ down-regulated, though differently, CD36, TSP-1 and CD61 transcripts.

Phagocytosis by macrophages is critical for the uptake and degradation of infectious agents and senescent cells, a process implicated in the negative regulation of immune responses and inflammatory processes in various organs [24]. Indeed, phagocytosis of apoptotic cells actively increases synthesis of immune-suppressive molecules (i.e. transforming growth factor-beta1, prostaglandin E2, and platelet-activating factor) thus inhibiting production of inflammatory cytokines (i.e IL-1, IL-8, tumor necrosis factor-alpha) in monocyte-derived macrophages [25]. Therefore, it is temping to speculate that deregulated clearance of apoptotic cells can contribute to sustain immune-inflammatory processes.

In conclusion, the present results indicate that CD-related inflammation is marked by diminished clearance of apoptotic cells/bodies, thus suggesting a role for such a defect in the ongoing mucosal inflammation in this disorder.
